# F-53B exposure accelerates progression from preexisting fatty liver to non-alcoholic steatohepatitis and hepatic fibrosis

**DOI:** 10.1016/j.isci.2026.115675

**Published:** 2026-04-10

**Authors:** Chunhua Hu, Liu Wu, Zehui Zhang, Yu Liu, Huihui Yang, Jian Zhou, Yongming Wu, Qiyu Wang

**Affiliations:** 1 School of Resources and Environment, Nanchang University, Nanchang 330031, China; 2 School of Water Resources & Environmental Engineering, East China University of Technology, Nanchang 330013, China; 3 Institute of Resources and Environment, Jiangxi Academy of Sciences, Nanchang 330012, China; 4 Department of Nephrology, Wuhan Children’s Hospital, Tongji Medical College, Huazhong University of Science & Technology, Wuhan 430000, China; 5 Institute of Medicine and Health, Jiangxi Academy of Sciences, Nanchang 330012, China

**Keywords:** biological sciences

## Abstract

6:2 Chlorinated polyfluoroalkyl ether sulfonate (F-53B) is prevalent in the environment, yet its effect on pre-existing metabolic dysfunction-associated steatotic liver disease (MASLD) remains unclear. Using adult male zebrafish with high-fat diet-induced MASLD, we investigated the hepatotoxicity of F-53B. Following exposure, F-53B exacerbated hepatic damage in MASLD zebrafish, accelerating progression to metabolic dysfunction-associated steatohepatitis (MASH) and fibrosis. Notably, environmentally relevant concentrations altered lipid metabolism and MASH/fibrosis markers only in MASLD fish, while high concentrations were required to affect normal-diet fish. This heightened susceptibility stemmed from increased hepatic F-53B accumulation in diseased livers. Mechanistically, liver-type fatty acid-binding protein (L-FABP) knockdown in HepG2 cells reduced lipid accumulation, inflammatory and fibrotic responses, identifying L-FABP as a key F-53B transporter. These findings demonstrate that metabolic dysfunction increases vulnerability to F-53B hepatotoxicity, highlighting the need to consider pre-existing conditions in environmental pollutant risk assessment.

## Highlights

• MASLD endows the higher total burden of F-53B in liver
• F-53B induces stronger hepatotoxicity in MASLD zebrafish vs. ND-fed
• F-53B exposure exacerbates preexisting fatty liver to MASH and fibrosis
• L-FABP knockdown attenuates F-53B-induced hepatotoxicity in HepG2 cells

## Graphical abstract

**Figure:**
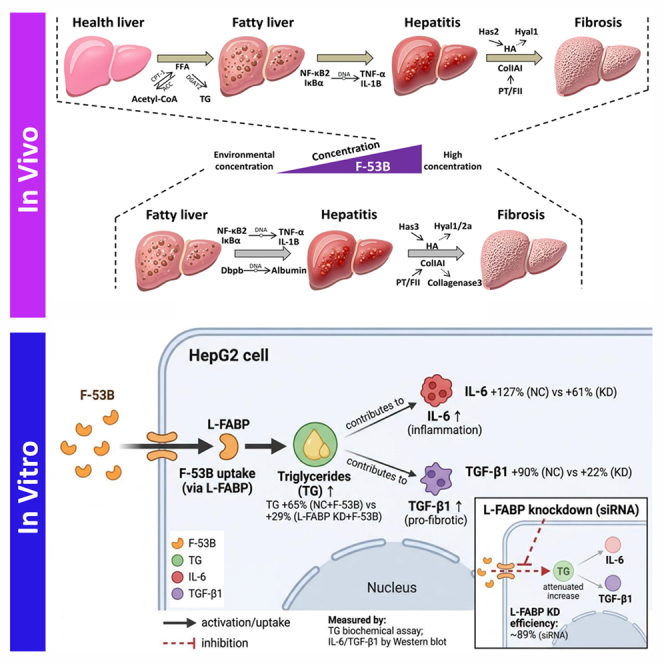

## Introduction

Per- and polyfluoroalkyl substances (PFAS), a class of anthropogenic chemicals that have been extensively utilized since the 1950s because of their unique physicochemical properties, have emerged as global contaminants due to their extreme environmental persistence and bioaccumulation potential.^1^^,^^2^ Perfluorooctane sulfonic acid (PFOS), a prominent PFAS, was classified as a persistent organic pollutant (POP) under the 2009 Stockholm Convention, which led to global restrictions on its production and use.^3^ Following the phasing out of PFOS, 6:2 chlorinated polyfluoroalkyl ether sulfonate (6:2 Cl-PFESA, commercial name F-53B) was adopted as the primary substitute in China’s electroplating industry.^4^^,^^5^ Between 2006 and 2015, the annual production of F-53B reached 10–14 tons in China, resulting in detectable environmental accumulation with concentrations ranging from 20 pg/L–112 μg/L in electroplating wastewater, sludge, and surface water.^6^^,^^7^^,^^8^ F-53B demonstrates significant bioaccumulation potential following environmental release, with tissue concentrations in freshwater and marine biota, as well as in human serum, often equaling or exceeding PFOS accumulation levels.^7^^,^^9^^,^^10^ F-53B has the longest half-life and poses the greatest risk to metabolic processes, posing health risks to both wildlife and humans.^11^^,^^12^ Additionally, the lack of specific restrictions on the emissions and disposal of F-53B further aggravated these adverse effects.

Over the years, F-53 biotoxicity has been widely studied because of its extensive distribution. A fatty liver is one of the main negative effects of F-53B.^11^ In male SD rats, F-53B administration markedly enhanced hepatic lipid droplet accumulation and upregulated proteins associated with both lipid synthesis and degradation.^13^ Additionally, F-53B exposure induced hepatic lipid degeneration and upregulated genes involved in fatty acid β-oxidation in C57BL/6 mice.^14^ Moreover, female C57BL/6 mice treated with F-53B developed hepatomegaly and cytoplasmic vacuolation in the liver, along with reduced acylcarnitine levels and disturbances in fatty acid transport.^15^ F-53B not only causes fatty liver in rodents but can also induce hepatic steatosis in zebrafish, as evidenced by increased liver index, vacuolation, hypertrophy within hepatic cells, elevated hepatic lipid content, and altered expression of lipid metabolism-related genes.^16^^,^^17^^,^^18^ Male zebrafish exhibit a more pronounced stress response in lipid metabolism than females following F-53B exposure.^19^ Although most studies have demonstrated that F-53B promotes the progression from normal liver function to fatty liver disease, the potential exacerbating effects of F-53B exposure on preexisting fatty liver conditions remain unexplored.

Metabolic dysfunction-associated steatotic liver disease (MASLD) is a common chronic liver disease prevalent in both developed and developing countries,^20^ with a global prevalence rate of 6%–35%.^21^^,^^22^ In the United States, 90 million people suffered from MASLD, accounting for 30% globally.^21^^,^^22^ MASLD can progress from simple steatosis to metabolic dysfunction-associated steatohepatitis (MASH); in more severe cases, it can progress to fibrosis, cirrhosis, and other serious liver complications.^23^^,^^24^ Research findings indicate that persistent exposure to organic pollutants, such as phenanthrene (Phe) and 2,3,7,8-tetrachlorodibenzo-p-dioxin (TCDD), contributes to the progression of MASLD to MASH and advanced fibrosis. For example, subcutaneous injection of environmentally relevant levels of Phe for 21 days significantly induced MASLD and MASH phenotypes, including reduced hepatosomatic index, dysregulation of PPARγ signaling and lipid metabolism, activation of the NF-κB pathway, and enhanced hepatic inflammatory responses, ultimately leading to liver fibrosis in newborn rats.^25^ Male C57BL/6J mice co-treated with a high-fat diet (HFD) and TCDD exhibited worsened hepatic steatosis, increased expression of several inflammation and fibrosis marker genes, and elevated liver collagen and serum transaminase levels compared to either HFD alone or a normal diet (ND) with TCDD exposure, indicating that TCDD accelerated liver fibrosis progression in MASLD mice.^26^ Currently, no study has determined whether F-53B promotes MASLD to a more severe end of the spectrum.

Based on these research gaps, the aim of this study was to examine whether F-53B exposure can exacerbate MASLD to more severe pathological stages, including MASH and hepatic fibrosis, in a zebrafish model of HFD-induced MASLD using molecular, biochemical, and histopathological analyses. Mechanistically, HepG2 cells, a suitable model for studying the progression from hepatic lipid disorders to inflammatory responses and fibrosis, will be utilized to investigate whether knockdown of liver-type fatty acid-binding protein (L-FABP), a key transporter of F-53B, reduces cellular uptake of F-53B and thereby attenuates inflammatory and fibrotic responses.^27^^,^^28^^,^^29^^,^^30^ To the best of our knowledge, this study addresses a knowledge gap regarding the potential exacerbating effects of F-53B exposure on preexisting fatty liver disease and its underlying mechanisms.

## Results

### HFD intervention induces MASLD in zebrafish

**Figure 1:**
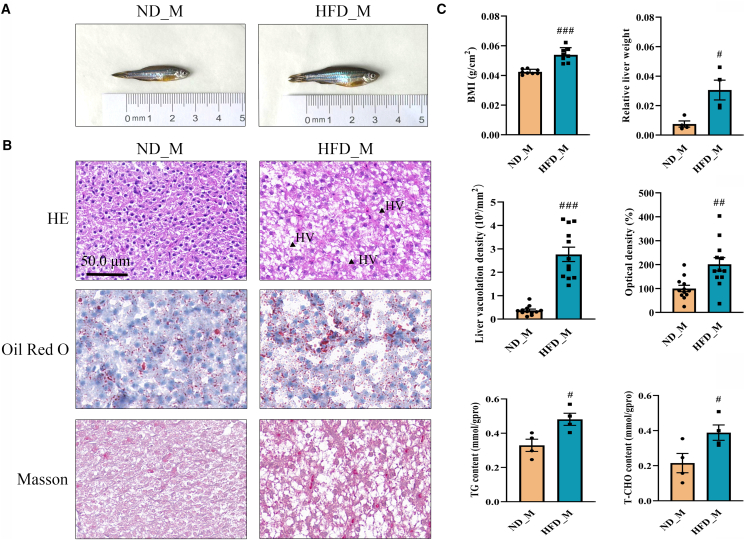
Successful metabolic dysfunction-associated steatotic liver disease zebrafish modeling (A) Images of zebrafish fed by a normal diet (ND_M) and high-fat diet (HFD_M) for 8-week. (B) Images of zebrafish liver sections from the ND_M and HFD_M groups following hematoxylin and eosin (H&E), Oil Red O (ORO), and Masson’s staining. HV indicates hepatic vacuoles. Scale bars, 50.0 μm. (C) Biochemical analysis of different model groups, including body mass index (BMI) (*n* = 8), relative liver weight (*n* = 4), liver vacuolation density in HE-stained sections (*n* = 12), optical density in ORO-stained sections (*n* = 12), and total triglyceride (TG) and total cholesterol (T-CHO) levels (*n* = 4) in hepatic tissue. Data are presented as mean ± SEM. The significance of differences between the ND_M and HFD_M groups was determined using the Mann-Whitney U test, and is indicated by hash symbols: ^#^*p* < 0.05, ^##^*p* < 0.01, ^###^*p* < 0.001.

HFD intervention significantly induced MASLD, as evidenced by a 27% increase in BMI, a 309% increase in relative liver weight, and nearly 7.5- and 2-fold greater accumulation of hepatic vacuoles and lipid droplets, respectively, in liver sections compared with those in the ND_M group (Figures 1A–1C). Biochemical analyses showed that HFD intervention significantly increased the levels of triglyceride (TG) and total cholesterol (T-CHO) in the liver by 46% and 81%, respectively, compared with those in the ND_M group (Figure 1C). However, HFD intervention did not lead to hepatic fibrosis (Figure 1B).

### Bioaccumulation of F-53B in ND- and HFD-treated zebrafish livers

**Figure 2:**
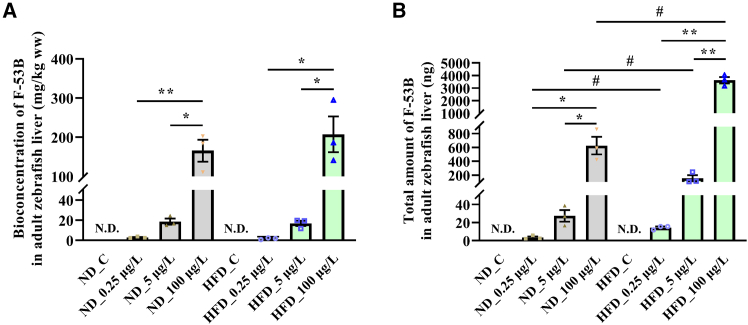
Higher total hepatic burden of F-53B in MASLD zebrafish (A) Bioconcentration of F-53B in the liver of zebrafish fed by normal diet (ND) and high-fat diet (HFD) following exposure to 0.25, 5, and 100 μg/L F-53B. (B) Total amount of F-53B in the liver of zebrafish fed by ND and HFD following exposure to 0.25, 5, and 100 μg/L F-53B. ND_C, the ND_M group was continued on ND without F-53B exposure. ND_0.25 μg/L, the ND_M group was continued on ND and further exposed to 0.25 μg/L F-53B for 28 days. ND_5 μg/L, the ND_M group was continued on ND and further exposed to 5 μg/L F-53B for 28 days. ND_100 μg/L, the ND_M group was continued on ND and further exposed to 100 μg/L F-53B for 28 days. HFD_C, the HFD_M group was continued on HFD without F-53B exposure. HFD_0.25 μg/L, the HFD_M group was continued on HFD and further exposed to 0.25 μg/L F-53B for 28 days. HFD_5 μg/L, the HFD_M group was continued on HFD and further exposed to 5 μg/L F-53B for 28 days. HFD_100 μg/L, the HFD_M group was continued on HFD and further exposed to 100 μg/L F-53B for 28 days. Data are presented as mean ± SEM (*n* = 3). Asterisks denote significant differences across F-53B exposure concentrations within the same diet group, analyzed by the Kruskal-Wallis test with Dunn’s post-hoc test (^∗^*p* < 0.05, ∗∗*p* < 0.01). Hash symbols denote significant differences between the ND and HFD groups at the same exposure concentration, analyzed by the Mann-Whitney U test (^#^*p* < 0.05).

F-53B levels in the liver of fish in the ND group following exposure to 0.25, 5, and 100 μg/L of F-53B were 2.6, 18.7, and 165.7 mg/kg ww (wet weight), respectively, whereas those in the HFD group were 1.8, 16.9, and 207.3 mg/kg ww, respectively (Figure 2A). Additionally, total amount of F-53B in the livers of zebrafish in the ND group following exposure to 0.25, 5, and 100 μg/L F-53B were 3.5, 27.4, and 625.1 ng, respectively, and 14.3, 154.1, and 3623.9 ng, respectively, in the HFD group (Figure 2B). F-53B was not detected in the ND_C or HFD_C groups (Figures 2A and 2B).

### F-53B exposure exacerbates liver damage in zebrafish model of MASLD

**Figure 3:**
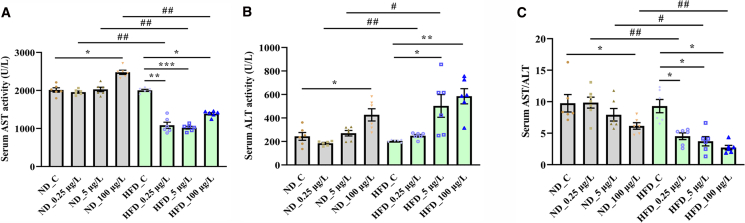
F-53B worsens liver damage in a zebrafish model of MASLD (A) The activity of aspartate aminotransferase (AST) in zebrafish serum in the ND and HFD groups following exposure to 0 (control), 0.25, 5, and 100 μg/L F-53B. (B) The activity of alanine aminotransferase (ALT) in zebrafish serum in the ND and HFD groups following exposure to 0 (control), 0.25, 5, and 100 μg/L F-53B. (C) The AST/ALT ratio determined in zebrafish serum in the ND and HFD groups following exposure to 0 (control), 0.25, 5, and 100 μg/L F-53B. Data are presented as mean ± SEM (*n* = 6). Asterisks denote significant differences across F-53B exposure concentrations within the same diet group, analyzed by the Kruskal-Wallis test with Dunn’s post-hoc test (^∗^*p* < 0.05, ∗∗*p* < 0.01, ∗∗∗*p* < 0.001). Hash symbols denote significant differences between the ND and HFD groups at the same exposure concentration, analyzed by the Mann-Whitney U test (^#^*p* < 0.05, ^##^*p* < 0.01).

Exposure to 100 μg/L F-53B significantly increased serum aspartate aminotransferase (AST) activity in the ND group (2477.8 U/L) compared with that in ND_C group (Figure 3A). Serum AST activities in the HFD group following exposure to 0.25, 5, and 100 μg/L F-53B were 1085, 1021.8, and 1387.9 U/L, respectively, which were markedly lower than that in the HFD_C group (2004.1 U/L; Figure 3A). Additionally, exposure to 100 μg/L F-53B increased serum alanine aminotransferase (ALT) activity in the ND group by 75% compared with that in the ND_C group (Figure 3B). Serum ALT activities in the HFD group increased in response to F-53B exposure, rising by approximately 146% and 186% at 5 and 100 μg/L, respectively, compared with the HFD_C group (Figure 3B). A modest, non-significant increase of 21% was observed at the 0.25 μg/L concentration in the HFD group compared with HFD_C group (Figure 3B). Serum AST/ALT ratio was lower in the ND_100 μg/L group than in the ND_C group (Figure 3C). Exposure to 0.25, 5, and 100 μg/L F-53B downregulated serum AST/ALT ratios in the HFD group compared with that in the HFD_C group (Figure 3C). Notably, there were significant differences in AST and ALT activities and AST/ALT ratios between the HFD and ND groups at each exposure concentration, except for ALT activity under 100 μg/L F-53B exposure (Figures 3A–3C).

### MASLD amplifies F-53B-induced lipid metabolic disorder

**Figure 4:**
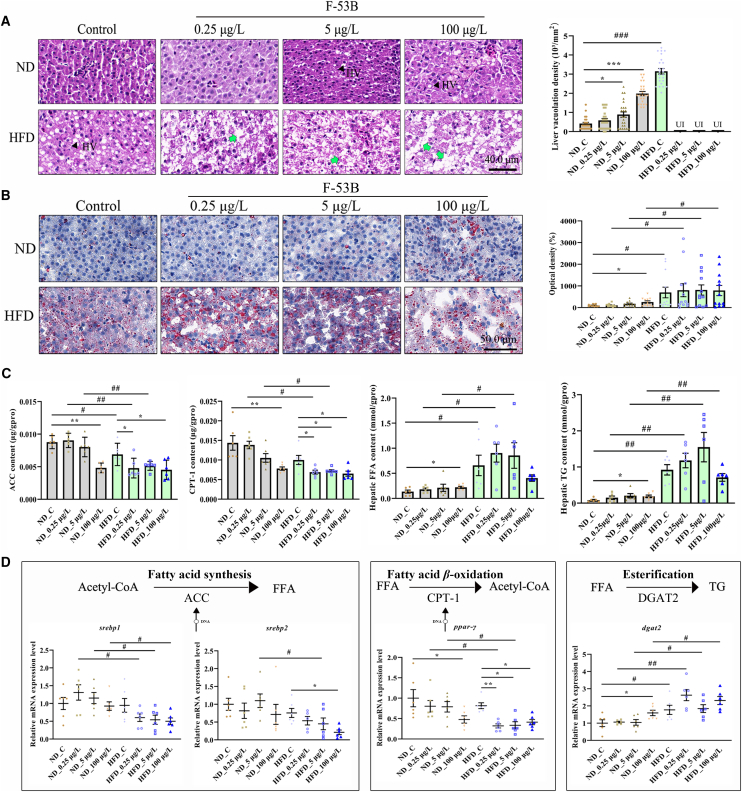
MASLD potentiates F-53B-induced lipid metabolic disorder (A) HE stained liver sections in the ND and HFD groups and corresponding liver vacuolation density (*n* = 24). HV, hepatic vacuoles; Green arrowheads indicate the lack of clear hepatic cord. Scale bars, 20.0 μm. (B) ORO-stained liver sections and corresponding optical density (*n* = 12). Scale bars, 25.0 μm. (C) Biochemical assessment of lipid metabolism indicators (*n* = 6), including the acetyl-CoA carboxylase (ACC), carnitine acyl-transferase-1 (CPT-1), free fatty acids (FFA), and TG. (D) Transcriptional levels of genes involved in regulating ACC and CPT-1 expression and fatty acid esterification (*n* = 6). Data are presented as mean ± SEM. Asterisks denote significant differences across F-53B exposure concentrations within the same diet group, analyzed by the Kruskal-Wallis test with Dunn’s post-hoc test (^∗^*p* < 0.05, ∗∗*p* < 0.01, ∗∗∗*p* < 0.001). Hash symbols denote significant differences between the ND and HFD groups at the same exposure concentration, analyzed by the Mann-Whitney U test (^#^*p* < 0.05, ^##^*p* < 0.01, ^###^*p* < 0.001).

Histopathological examination showed that hepatic vacuolation density increased with increasing F-53B exposure concentrations in the ND-treated groups. Specifically, vacuolation density was significantly higher in the ND group than in the ND_C group following exposure to 5 and 100 μg/L F-53B (Figure 4A). The vacuolation density was higher in the HFD_C group than in the ND_C group; however, this could not be determined in MASLD livers under F-53B exposure owing to the lack of a clear hepatic cord (Figure 4A). Oil Red O (ORO) staining showed that there was an increase in lipid accumulation in the ND_100 μg/L group and all HFD groups, but there was no significant difference between the HFD groups (Figure 4B). Moreover, ORO staining intensity was significantly higher in the HFD group than in the ND group at all exposure concentration (Figure 4B). Biochemical analysis showed that there was a decrease in the hepatic levels of acetyl-CoA carboxylase (ACC) and carnitine acyl-transferase-1 (CPT-1) and an increase in the hepatic levels of free fatty acid (FFA) and TG in the ND_100 μg/L group, and there were significant differences in the levels of all indicators between the HFD groups and ND groups at all exposure concentrations, except for the ACC under 100 μg/L exposure and CPT-1 under control and 100 μg/L exposure (Figure 4C). F-53B exposure significantly decreased ACC and CPT-1 levels in the HFD group compared with the HFD_C group (Figure 4C). However, the reduction in ACC was not significant in the HFD_5 μg/L group (Figure 4C). Additionally, there were no significant differences in FFA and TG levels among the HFD groups (Figure 4C). We examined the mRNA expression of genes involved in regulating ACC, CPT-1 expression, and fatty acid esterification. *srebp1* mRNA levels were lower in the HFD group than in the ND group at all F-53B exposure levels (Figure 4D). *srebp2* mRNA expression was significantly lower in HFD_100 μg/L and HFD_5 μg/L groups than in HFD_C and ND_5 μg/L groups, respectively (Figure 4D). *ppar-γ* and *dgat2* expression patterns were similar to those of CPT-1 and TG, respectively (Figures 4C and 4D).

### F-53B exposure promotes MASLD to hepatitis

**Figure 5:**
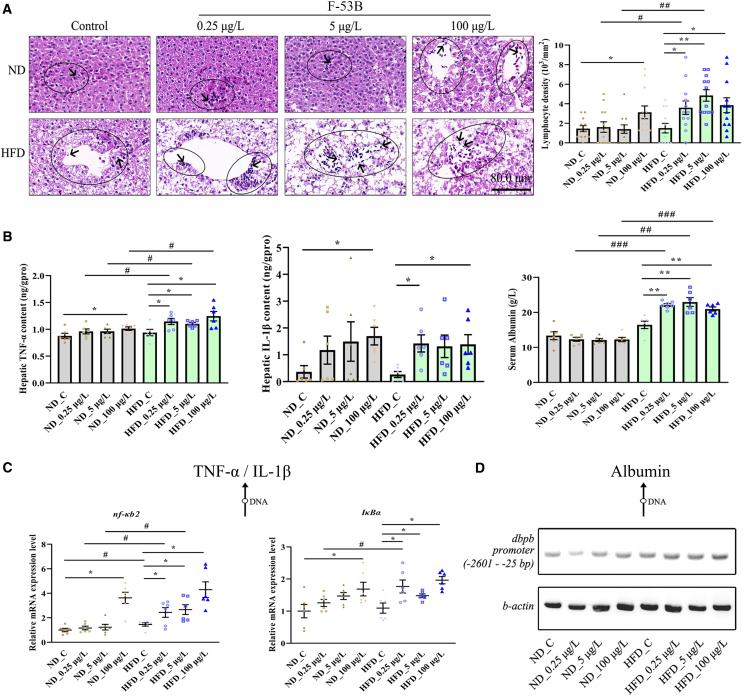
F-53B exposure accelerated the progression of hepatitis (A) Assessment of lymphocyte infiltration and density in HE-stained liver sections (*n* = 12). Black circles indicate inflammatory cell infiltration; Black arrows indicate the lymphocytes. Scale bars, 40.0 μm. (B) Measurements of inflammation-related indicators (*n* = 6), including the hepatic tumor necrosis factor-α (TNF-α), hepatic interleukin-1β (IL-1β), and serum albumin. (C) Detection of genes associated with inflammatory responses (*n* = 6), including *NF-κB2* and *IκBα*. (D) Semi-quantitative PCR of *dbpb* expression (*n* = 5), which is associated with albumin synthesis. Data are presented as mean ± SEM. Asterisks denote significant differences across F-53B exposure concentrations within the same diet group, analyzed by the Kruskal-Wallis test with Dunn’s post-hoc test (^∗^*p* < 0.05, ∗∗*p* < 0.01). Hash symbols denote significant differences between the ND and HFD groups at the same exposure concentration, analyzed by the Mann-Whitney U test (^#^*p* < 0.05, ^##^*p* < 0.01, ^###^*p* < 0.001).

F-53B exposure significantly induced hepatic lymphocyte accumulation, with levels markedly increased in the ND_100 μg/L group and all F-53B-exposed HFD groups compared with ND_C and HFD_C groups, respectively; furthermore, lymphocyte levels in the HFD_0.25 and HFD_5 μg/L groups were significantly higher than in their corresponding ND groups (Figure 5A). Hepatic levels of tumor necrosis factor-α (TNF-α) and interleukin-1β (IL-1β) were significantly elevated in response to F-53B exposure. Specifically, both cytokines were higher in the ND_100 μg/L group than in the ND_C group, and in all F-53B-exposed HFD groups compared to the HFD_C group, with the exception of IL-1β in the HFD_5 μg/L group (Figure 5B; Table S1). Furthermore, at every F-53B concentration, TNF-α levels were consistently higher in the HFD group than in the corresponding ND group (Figure 5B; Table S1). Exposure to 0.25, 5, and 100 μg/L F-53B significantly increased serum albumin levels compared with those in the HFD_C and ND groups (Figure 5B; Table S1). We also examined the mRNA expression of TNF-α, IL-1β, and albumin synthesis-related genes. Quantitative real-time PCR (real-time qPCR) showed that *NF-κB2* expression showed a similar trend to TNF-α expression pattern, with only slight variations (Figures 5B and 5C). The expression of the *NF-κB2* inhibitor *IκBα* was significantly up-regulated upon F-53B exposure. It was higher in the ND_100 μg/L group than in the ND_C group, and in all F-53B-exposed HFD groups than in the HFD_C group (Figure 5C). *IκBα* mRNA expression was additionally higher in the HFD_0.25 μg/L group than in the ND_0.25 μg/L group (Figure 5C). Semi-quantitative PCR showed an increase in the abundance of the *dpbp* promoter located between −2601 and −25 bp in F-53B-exposed HFD groups, which was similar to the level of serum albumin (Figures 5B and 5D).

### F-53B exposure accelerates MASLD progression to hepatic fibrosis

**Figure 6:**
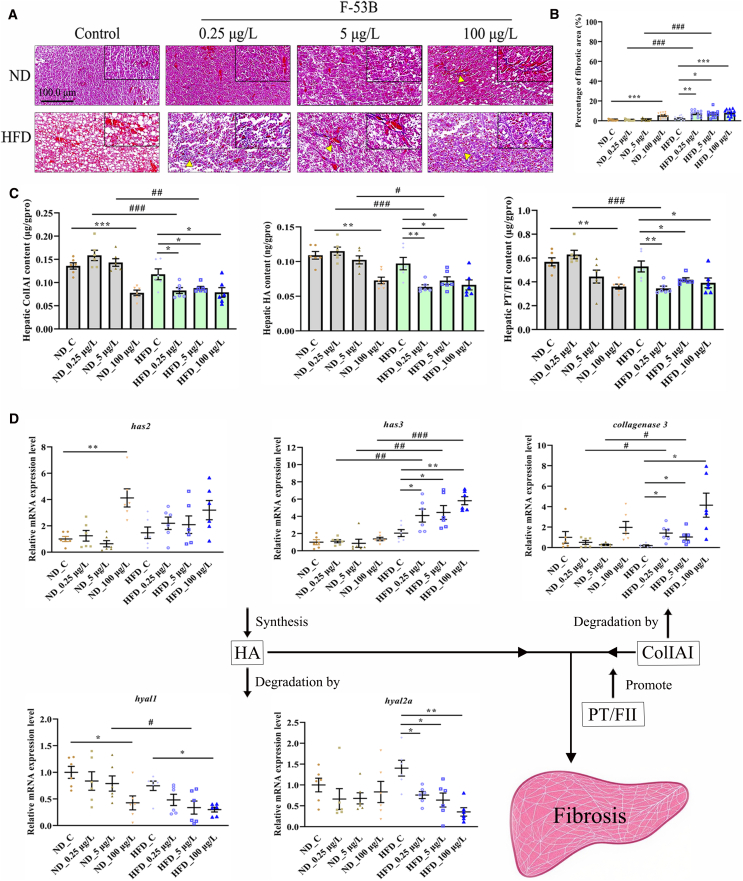
F-53B exposure accelerated the transition from MASLD to fibrosis (A) Representative Masson’s staining of liver sections, with a magnified view of collagen deposition in a fibrotic area. Yellow arrowheads indicate the fiber. Scale bars, 50.0 μm. (B) Percentage of fibrotic area in liver sections in the ND and HFD groups following F-53B exposure (*n* = 12). (C) Determination of the expression of the fibrosis-related indicators in liver (*n* = 6), including the collagen IAI (ColIAI), HA, and prothrombin time/factor II (PT/FII). (D) Detection of genes involved in HA synthesis and degradation and ColIAI degradation (*n* = 6). Data are presented as mean ± SEM. Asterisks denote significant differences across F-53B exposure concentrations within the same diet group, analyzed by the Kruskal-Wallis test with Dunn’s post-hoc test (^∗^*p* < 0.05, ∗∗*p* < 0.01, ∗∗∗*p* < 0.001). Hash symbols denote significant differences between the ND and HFD groups at the same exposure concentration, analyzed by the Mann-Whitney U test (^#^*p* < 0.05, ^##^*p* < 0.01, ^###^*p* < 0.001).

Masson’s staining revealed hepatic fibrosis in the ND_100 μg/L, HFD_0.25 μg/L, HFD_5 μg/L, and HFD_100 μg/L groups, despite the absence of massive fiber accumulation (Figures 6A and 6B). Importantly, there was a significant decrease in the expression of the fibrosis-related indicators collagen IAI (ColIAI), hyaluronic acid (HA), and prothrombin time/factor II (PT/FII) in the livers of HFD_0.25 μg/L, HFD_5 μg/L, and HFD_100 μg/L groups compared with that in the HFD_C group (Figure 6C; Table S2). Additionally, the levels of all fibrosis-related indicators were lower in the livers of ND_100 μg/L group than in the ND_C group (Figure 6C; Table S2). Moreover, hepatic ColIAI and HA levels were lower in the HFD_0.25 μg/L and HFD_5 μg/L groups than in their corresponding ND groups (Figure 6C; Table S2). Hepatic PT/FII level was lower in the HFD_0.25 μg/L group than in the ND_0.25 μg/L group (Figure 6C; Table S2). The mRNA expression of HA synthesis- and degradation-related genes, as well as ColIAI degradation gene were also examined. *has2* mRNA expression was higher in the ND_100 μg/L group than in the ND_C group, and exposure to all concentrations of F-53B increased *has3* expression in all HFD groups compared with those in the HFD_C and ND groups (Figure 6D). Although both *hyal1* and *hyal2a* were downregulated in the HFD group after F-53B exposure, their expression patterns differed. Specifically, *hyal2a* was significantly downregulated across all F-53B-exposed HFD groups compared to the HFD_C group, whereas *hyal1* showed a less consistent reduction (Figure 6D). The level of *collagenase 3* mRNA were significantly higher in the HFD_0.25 μg/L and HFD_5 μg/L groups than in the HFD_C group and their corresponding ND groups, and its expression in HFD_100 μg/L group was only higher than in HFD_C group (Figure 6D).

### Knockdown of L-FABP alleviated F-53B-induced inflammatory and fibrotic responses in HegG2 cells

**Figure 7:**
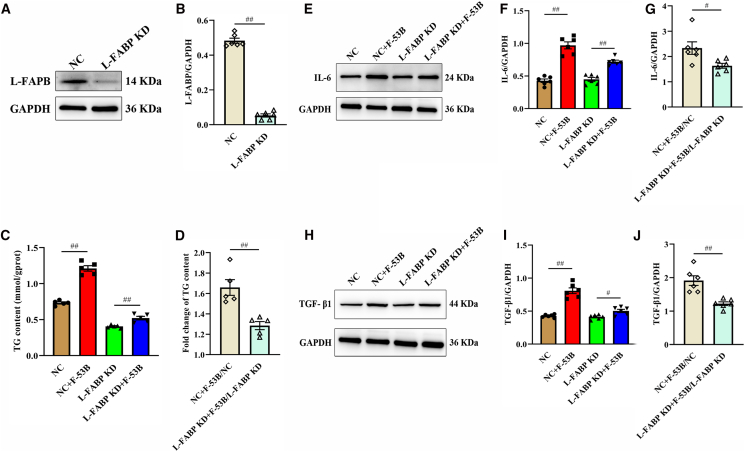
Knockdown of L-FABP mitigated F-53B-induced damage in HepG2 cells (A) Liver-type fatty acid-binding protein (L-FABP) knockdown was confirmed by immunoblot analysis using glyceraldehyde-3-phosphate dehydrogenase (GAPDH) as the loading control (*n* = 6). (B) Calculated L-FABP/GAPDH ratio (*n* = 6) based on the immunoblot results shown in (A). (C) Effects of different treatments on TG content in HepG2 cells (*n* = 5). (D) Fold change of TG content in NC + F-53B/NC and L-FABP KD + F-53B/L-FABP KD groups (*n* = 5). (E) Interleukin-6 (IL-6) expression in HepG2 cells following different treatments. (F) Relative IL-6 expression (IL-6/GAPDH) (*n* = 6). (G) Fold change of IL-6 (relative to respective control) in NC vs. L-FABP KD groups with F-53B treatment (*n* = 6). (H) Transforming growth factor β1 (TGF-β1) expression in HepG2 cells following different treatments. (I) Relative TGF-β1 expression (TGF-β1/GAPDH) (*n* = 6). (J) Fold change of TGF-β1 (relative to respective control) in NC vs. L-FABP KD groups with F-53B treatment (*n* = 6). NC, negative control siRNA group. NC + F-53B, NC with 5 mg/L F-53B group. L-FABP KD, L-FABP-knockdown group. L-FABP KD + F-53B, L-FABP KD with 5 mg/L F-53B group. Data are presented as mean ± SEM. The significance of differences between two groups was determined using the Mann-Whitney U test, and is indicated by hash symbols: ^#^*p* < 0.05, ^##^*p* < 0.01.

L-FABP expression was successfully knocked down by siRNA, resulting in an 89% reduction compared to the NC control (Figures 7A and 7B; Figure S1). NC and L-FABP KD cells were treated with or without F-53B, followed by collection of cell lysates for quantification of TG, interleukin-6 (IL-6), and transforming growth factor β1 (TGF-β1). F-53B treatment increased TG content by 65% in NC cells and by 29% in L-FABP KD cells, with the increase being significantly greater in the NC group (Figures 7C and 7D). Quantification of IL-6 showed that F-53B treatment increased IL-6 expression by 127% in NC cells and 61% in L-FABP KD cells, as quantified by western blot, and the induction was significantly stronger in the NC group (Figures 7E–7G; Figure S2). TGF-β1 expression exhibited a trend similar to that of IL-6, increasing by 90% in NC cells and 22% in L-FABP KD cells after F-53B treatment, with a significantly greater change observed in the NC group (Figures 7H–7J; Figure S3).

## Discussion

F-53B is an emerging PFOS substitute with documented hepatotoxic potential, primarily studied for its ability to induce steatosis in healthy models.^15^^,^^17^ However, its impact on the trajectory of pre-existing metabolic liver disease, a scenario of greater clinical and public health relevance, remains unknown. Our study directly addresses this critical gap and provides three key advances. First, we presented the experimental evidence that environmentally relevant concentrations of F-53B accelerated the progression from HFD-induced MASLD to advanced pathological stages, including MASH and hepatic fibrosis, in an adult male zebrafish model. Second, we identified a pivotal mechanism underlying this heightened susceptibility of a significantly higher total hepatic burden of F-53B in metabolically compromised livers, which was verified by the fact that L-FABP knockdown in HepG2 cells attenuated hepatic lipid accumulation and its progression to hepatitis and fibrosis. Third, through an integrated analysis of histopathology, biochemistry, and gene expression, we delineated a coherent pathophysiological pathway linking increased pollutant accumulation to dysregulated lipid metabolism, amplified inflammatory response, and the activation of fibrogenesis. The following discussion interprets our findings within this conceptual framework.

The liver serves as the primary site for F-53B enrichment, and its accumulation in hepatic tissues is a key factor in its hepatotoxicity.^9^^,^^19^^,^^29^ To assess the effect of different health conditions on F-53B bioaccumulation, we quantified F-53B levels in the zebrafish livers of HFD and ND groups. Neither ND nor HFD significantly influenced F-53B bioconcentration in the liver at all exposure concentrations. However, the total hepatic burden of F-53B was notably higher in zebrafish with MASLD than those in the ND group, which could be attributed to the increased liver weight associated with MASLD. Overall, these findings suggest that F-53B exposure may exert more pronounced adverse effects on the livers of zebrafish with HFD-induced MASLD than those in the ND group.

To validate our hypothesis, we assessed hepatic function by measuring key biomarkers of liver injury. AST and ALT levels are well-established indicators of liver function, and their abnormal activity or altered AST/ALT ratios signify hepatic damage, including metabolic dysfunction, inflammation, and fibrosis.^3^^,^^31^ Therefore, we examined serum AST and ALT activities and AST/ALT ratio in the ND and HFD groups under exposure to varying concentrations of F-53B. Hepatic injury only occurred in zebrafish in ND group following exposure to the highest concentration of F-53B (100 μg/L). In contrast, zebrafish with HFD-induced MASLD exhibited liver damage following exposure to all concentrations of F-53B (0.25–100 μg/L), showing significantly greater severity than those in the ND group at each corresponding exposure level. Collectively, these findings suggest that MASLD exacerbates the susceptibility to F-53B-induced hepatotoxicity, rendering the liver more vulnerable to functional impairment even at relatively low concentrations. Similarly, preexisting MASLD has been shown to enhance perfluorooctanoic acid (PFOA)-induced lipid peroxidation pathways in male C57BL/6 mice, and HFD intake and phthalate exposure resulted in amplified hepatotoxic effects.^32^^,^^33^ These observations in our model highlight the potential for synergistic health risks arising from the interaction between metabolic dysfunction and pollutant exposure. Notably, in the present study, the HFD_C group exhibited clear histopathological and biochemical hallmarks of MASLD, yet serum AST and ALT levels did not differ significantly from the ND_C group, a pattern aligned with reports that MASLD can occur without significant transaminase alterations, likely reflecting a compensated metabolic state prior to overt hepatocyte damage.^34^^,^^35^^,^^36^^,^^37^ Transaminase elevation is not an inevitable marker of simple steatosis in zebrafish MASLD models. Simultaneously, the divergent responses in the profile of serum AST levels between ND and HFD groups following F-53B exposure indicate that the pathological response to this pollutant is fundamentally altered in a metabolically compromised liver.

Subsequently, each stage of liver disease was systematically assessed, beginning with an investigation into the exacerbating effects of F-53B on lipid metabolism in preexisting fatty liver conditions. Hematoxylin and Eosin (H&E) and ORO staining showed that F-53B did not exacerbate lipid accumulation in the liver of zebrafish with HFD-induced MASLD compared with that in the HFD_C group. However, the liver of zebrafish with HFD-induced MASLD exhibited disrupted hepatic cord structure following F-53B exposure. Consistent with previous findings,^16^^,^^19^ hepatic steatosis was observed in fish in the ND group only at the highest F-53B exposure concentration of 100 μg/L. Biochemical analysis indicated that HFD treatment significantly altered all lipid metabolism related indicators, including ACC, CPT-1, FFA, and TG, compared with those in the ND groups across all F-53B exposure concentrations, except for ACC and CPT-1 levels in the 100 μg/L exposure group. Consistent ACC and CPT-1 expression patterns between ND_100 μg/L and HFD_100 μg/L groups indicated that lipid metabolic dysregulation was mainly caused by exposure to high concentrations of F-53B and not MASLD. Similar dominance was also observed in subsequent MASH and fibrosis progression analyses, including parameters, such as lymphocyte density and IL-1β, ColIAI, HA, and PT/FII levels. Notably, only the highest F-53B concentration (100 μg/L) altered hepatic ACC and CPT-1 levels in zebrafish in the ND group. In contrast, with the exception of ACC in the HFD_5 μg/L group, nearly all tested F-53B concentrations altered these markers in zebrafish with HFD-induced MASLD relative to the HFD_C group. Overall, these results indicated that MASLD potentiated F-53B-induced hepatic lipid dysregulation. Regarding the stable hepatic FFA and TG levels across F-53B concentrations in HFD groups, this likely reflects a metabolic plateau induced by the diet itself, where hepatic lipid storage was near saturation. In this state, the key effect of F-53B shifts from quantitatively increasing lipids to qualitatively driving pathology from steatosis into inflammatory and fibrotic stages. Thus, the persistently high FFA/TG represents the pre-existing metabolic burden, upon which F-53B acts to promote MASH and fibrosis, as supported by the marked inflammatory and fibrotic responses.

Building on our biochemical data, a plausible mechanistic explanation is that accumulated FFA may have contributed to the increase in TG through esterification, and the reduced CPT-1 level may have suppressed the transition from fatty acid into acetyl-CoA by inhibiting fatty acid β-oxidation, further exacerbating TG accumulation. Conversely, ACC downregulation, which is the rate-limiting step in *de novo* lipogenesis, may help curb excess TG production by attenuating fatty acid synthesis from acetyl-CoA. Therefore, we examined the mRNA levels of genes involved in regulating ACC and CPT-1 transcription and fatty acid esterification, including *srebp1*/2, *ppar-γ*, and *dgat2*. *srebp1* and *srebp2* downregulation contributed to decreased ACC expression in the ND_100 μg/L and all F-53B-exposed HFD groups. The consistent expression patterns between *ppar-γ* and CPT-1, as well as between *dgat2* and TG levels, supported the consistency of the biochemical profile.

Previous studies have shown that F-53B can induce MASH in mice and zebrafish, which was accompanied by significant changes in pro-inflammatory cytokines, with NF-κB mediating these immune responses via direct binding to F-53B.^14^^,^^38^^,^^39^ Our study further confirmed that exposure to high concentrations of F-53B promoted lymphocyte infiltration, increased pro-inflammatory cytokine levels, and upregulated *NF-κB2* expression. Importantly, the preexisting MASLD exacerbated this immune response, as manifested in the fact that exposure to environmental concentrations of F-53B induced the expression of *NF-κB2* and upregulated IL-1β and TNF-α levels. The progression to MASH was simultaneously associated with further upregulation of *IκBα* expression, which could attenuate the inflammatory responses by suppressing the activity of dimeric NF-κB/REL complexes.^40^ Additionally, we examined the levels of albumin, a biomarker of MASH,^41^ and observed a significant increase in albumin concentration exclusively in the F-53B-exposed HFD groups. Semi-quantitative PCR demonstrated increased *dbpb* replication levels in all F-53B-exposed HFD groups, consistent with its role as a transcriptional regulator of albumin levels.^42^ It is important to note that the semi-quantitative PCR used to assess *dbpb* provides a comparative measure of promoter-region amplification rather than a direct, quantitative mRNA expression level. While this approach offered a reproducible signal that correlated with albumin changes, its interpretation as a proxy for transcriptional activity should be considered within this methodological context. Overall, these results confirmed that F-53B exposure accelerated MASLD progression to a more serious outcome.

Beyond driving MASH, F-53B exposure additionally promoted hepatic fibrosis development in the ND_100 μg/L group and all F-53B-exposed HFD groups, as evidenced by increased accumulation of fiber in liver sections. Consistent with the observed fibrotic phenotype, the exposed groups showed decreased levels of soluble extracellular matrix components (ColIAI and HA) and coagulation factors (PT/FII) in liver tissues. This apparent discrepancy, where active fibrogenesis coincides with a reduction in soluble markers, is explained by our assay methodology. As detailed in the method details, liver tissues were homogenized in PBS, which efficiently extracts soluble proteins but not insoluble polymeric structures. Therefore, the observed reduction in soluble ColIAI, HA, and PT/FII levels directly reflects their active polymerization and incorporation into insoluble fibrillar deposits during the fibrogenic process. Overall, the progression to fibrosis followed a pattern analogous to MASH development under F-53B exposure, wherein preexisting MASLD exacerbated fibrogenesis, which was manifested by the environmentally relevant concentration of F-53B was sufficient to trigger hepatic fibrosis. Importantly, F-53B has been shown to also induce fibrosis in mouse kidney.^43^ At the molecular level, the upregulation of *has2/3* coupled with the downregulation of *hyal1/2a* promoted HA accumulation by enhancing HA synthesis and impairing HA degradation.^44^ The synergistic action of HA, ColIAI, and PT/FII ultimately drove hepatic fibrogenesis, with PT/FII implicated in mediating Col1A1 cross-linking.^45^ Elevated *collagenase 3* expression likely functioned to reduce ColIAI accumulation and mitigate fibrosis progression.^46^ In brief, F-53B exposure was associated with the progression to liver fibrosis on the basis of MASH.

To investigate the underlying mechanisms, HepG2 cells, a suitable model for studying the progression from hepatic lipid disorders to inflammatory and fibrotic responses, were utilized in this study.^27^^,^^47^^,^^48^ L-FABP serves as a key transporter for F-53B uptake from extracellular into intracellular compartments in hepatic cells.^28^^,^^29^ L-FABP was selected for siRNA-mediated knockdown to determine whether reducing its expression could decrease cellular uptake of F-53B to attenuate inflammatory and fibrotic responses. Results showed that knockdown of L-FABP attenuated F-53B-induced lipid accumulation, inflammatory responses, and fibrosis. This was supported by a weaker induction of TG content, inflammatory factor IL-6, and pro-fibrotic factor TGF-β1 levels compared to that in F-53B-treated NC cells. Our *in vitro* experiments demonstrated that inhibiting F-53B uptake attenuated hepatic lipid accumulation and its progression to hepatitis and fibrosis. This finding aligned with the observed high enrichment of F-53B in fatty liver of zebrafish, as identified in our earlier analysis.

To our knowledge, this study offers insights from a zebrafish model, showing that F-53B accelerates the transition from MASLD to more severe pathological stages. MASLD exacerbated the susceptibility to F-53B-induced hepatotoxicity, as evidenced by the amplified lipid metabolic disorders and accelerated progression to a more severe end of the spectrum, such as MASH and hepatic fibrosis. The progression from MASLD to more severe stages may be associated with the higher total burden of F-53B in the MASLD liver, which was also verified by *in vitro* assay. Notably, some negative effects were associated with exposure to high concentrations of F-53B but not with MASLD. The adult zebrafish model used here recapitulates key human MASLD features of steatosis, inflammation, and fibrosis, via conserved pathways in lipid metabolism, inflammation, and fibrogenesis, making it valuable for probing core mechanisms of pollutant-aggravated disease. However, interspecies differences in anatomy, immune complexity, and xenobiotic metabolism necessitate caution in direct extrapolation to human MASH and fibrosis. Thus, while our study robustly identifies how pre-existing metabolic dysfunction amplifies F-53B hepatotoxicity, future mammalian studies are needed to fully assess its human relevance. Our findings underscore the need to consider differential susceptibility among individuals with varying metabolic health states to environmental pollutants.

### Limitations of the study

This study has several limitations. First, only male zebrafish were used. This choice was based on evidence that males exhibit greater hepatic metabolic vulnerability and more pronounced lipid dysregulation upon F-53B exposure,^19^ which was consistent with our aim to investigate pollutant-aggravated injury in a susceptible system. Consequently, our findings may not be generalizable to females, and future work should examine potential sex-specific effects. Second, the sample size was determined based on established protocols in zebrafish toxicology rather than a formal *a priori* power calculation. Although the sample size proved sufficient to detect the significant effects reported, *a priori* calculation remains a best practice for optimizing experimental design. Third, the assessment of steatosis progression included the quantification of hepatic vacuolation density. Notably, this specific metric did not show a further increase in HFD groups upon F-53B exposure. We interpret that this was due to a fundamental shift in pathology: the pollutant’s effect in the context of MASLD was not to augment lipid accumulation per se but to drive a transition toward more advanced injuries. This progression was characterized by significant disruption of hepatic cord architecture and fusion of lipid vacuoles, which rendered precise vacuole counting challenging and shifted the primary pathology toward inflammation and fibrogenesis. Consequently, while vacuolation density quantified the initial steatosis, its limited change in this context underscores that this metric alone may have insufficient sensitivity to capture a qualitative transition in the nature of liver damage. Future studies could therefore complement such histological measures with more dynamic biochemical assessments of lipid metabolism. Finally, although lymphocyte infiltration in HE-stained liver sections was used as an indicator of inflammation, this single parameter is insufficient to comprehensively characterize MASH. Given the complexity of MASH pathology, future studies should incorporate additional molecular markers or validated histological scoring systems to more accurately capture the progression to MASH.

## Resource availability

### Lead contact

Further information and requests for resources and reagents should be directed to the lead contact, Qiyu Wang (wangqiyu@jxas.ac.cn).

### Materials availability

This study did not generate new unique reagents or cell lines.

### Data and code availability

• All data supporting the findings of this study are found within the article and its supplemental information.
• This article does not report the original code.
• Any additional information about the data reported in this paper will be shared by the lead contact upon request.

## Acknowledgments

This research work was financially supported by the 10.13039/501100001809National Natural Science Foundation of China (82502259), 10.13039/501100004479Jiangxi Provincial Natural Science Foundation (20242BAB25201 and 20252BAC250157), Jiangxi Provincial Science and Technology Plan Project (20232BCJ22047 and 20252BCF320026), and Innovation Platform for Hardware Facilities Construction of 10.13039/501100014868Jiangxi Academy of Sciences (2024CXPT1002).

## Author contributions

C.H. and L.W. contributed to conceptualization, data curation, formal analysis, investigation, methodology, and writing – original draft; C.H., Z.Z., Y.L., and H.Y. contributed to resources, software, validation, and visualization; H.Y. and J.Z. contributed to writing – review and editing; Y.W. contributed to supervision and funding acquisition; Q.W. contributed to funding acquisition, project administration, and supervision. All authors read and approved the final manuscript.

## Declaration of interests

The authors have filed a patent application related to the findings of this study (application no. 2025106269382, filed on 5/15/2025 to China National Intellectual Property Administration (CNIPA) as inventors.

## Declaration of generative AI and AI-assisted technologies in the writing process

During the preparation of this work the authors used DeepSeek (https://chat.deepseek.com/) for grammatical corrections and sentence refinements. After using this tool/service, the authors reviewed and edited the content as needed and take full responsibility for the content of the published article.

## STAR★Methods

### Key resources table

**Table:** 

REAGENT or RESOURCE	SOURCE	IDENTIFIER
**Antibodies**		

L-FABP	Santa Cruz Biotechnology	sc-271591; RRID: AB_10650273
IL-6	Proteintech	21865-1-AP; RRID: AB_11142677
TGF-β1	Proteintech	81746-2-RR; RRID: AB_3670503
GAPDH	Proteintech	10494-1-AP; RRID: AB_2263076
HRP-conjugated Goat Anti-Mouse IgG(H + L)	Proteintech	SA00001-1; RRID: AB_2722565
HRP-conjugated Goat Anti-Rabbit IgG(H + L)	Proteintech	SA00001-2; RRID: AB_2722564

**Biological samples**		

Zebrafish liver	This Paper	N/A
Zebrafish serum	This Paper	N/A

**Chemicals, peptides, and recombinant proteins**		

F-53B	Shanghai Maikun Chemical Co., Ltd.	CAS No. 73606-19-6
DMSO	Sigma-Aldrich	34869
DMEM	Gibco	11995040
FBS	Gibco	10099-141C
Penicillin-streptomycin	Sigma-Aldrich	V900929
MS-222	Sigma-Aldrich	E10521
13C4-labeled L-PFOS	Wellington Laboratories	MPFOS

**Critical commercial assays**		

TG	Nanjing Jiancheng Bioengineering Institute	A110-1-1
T-CHO	Nanjing Jiancheng Bioengineering Institute	A111-1-1
ACC	Nanjing Jiancheng Bioengineering Institute	H232-1-2
CPT-1	Nanjing Jiancheng Bioengineering Institute	H230-1-2
FFA	Nanjing Jiancheng Bioengineering Institute	A042-2-1
TNF-α	Nanjing Jiancheng Bioengineering Institute	H052-1-2
IL-1β	Nanjing Jiancheng Bioengineering Institute	H002-1-2
ColIAI	Nanjing Jiancheng Bioengineering Institute	H142-1-1
HA	Nanjing Jiancheng Bioengineering Institute	H141-1-2
PT/FII	Nanjing Jiancheng Bioengineering Institute	H345-1-2
AST	Nanjing Jiancheng Bioengineering Institute	C010-2-1
ALT	Nanjing Jiancheng Bioengineering Institute	C009-2-1
PrimeScript™ RT reagent Kit with gDNA Eraser	Takara	RR047A
TB Green Premix Ex Taq II (li RNaseH Plus)	Takara	RR420B (A×2)
L-FABP siRNA (h)	Santa Cruz Biotechnology	sc-41243
siRNA Transfection Reagent	Santa Cruz Biotechnology	sc-29528
siRNA Transfection Medium	Santa Cruz Biotechnology	sc-36868
siRNA Dilution Buffer	Santa Cruz Biotechnology	sc-29527
Control siRNA	Santa Cruz Biotechnology	sc-37007
RIPA	Beyotime	P0013K
DNAzol	Thermo Fisher Scientific	10503027
BCA assay kit	Beyotime	P0009
TransTaq DNA polymerase (HiFi)	TransGen Biotech	AP131

**Experimental models: Cell lines**		

HepG2	ATCC	HB-8065

**Experimental models: Organisms/strains**		

Zebrafish	China Zebrafish Resource Center	AB

**Oligonucleotides**		

qRT-PCR	This Paper	Table S4
Semi-quantitative PCR	This Paper	Table S5

**Software and algorithms**		

GraphPad Prism 9.0	GraphPad	https://www.graphpad.com/resources
ImageJ 1.54	ImageJ	https://imagej.nih.gov/ij/

### Experimental model and study participant details

#### Zebrafish maintenance

Three-month-old male wild-type adult zebrafish (AB strain) were maintained in a zebrafish aquarium facility (Tecniplast Zebtec, Tecniplast, Buguggiate, Italy) at 27 ± 1 °C under a 14 h light/10 h dark photoperiod. All experimental procedures involving animals were approved by the Independent Animal Care and Use Committee of Jiangxi Academy of Sciences (Approval No. 2023-051) and conformed to the relevant institutional and national guidelines. Only male fish were used in this study to control for variability associated with sex, and consequently, the results reflect responses specific to males. The influence of sex on F-53B hepatotoxicity in the context of MASLD remains to be determined.

#### Cell culture

HepG2 human hepatocellular carcinoma cells (ATCC, HB-8065) were maintained in a humidified incubator at 37 °C with 5% CO_2_. Cells were cultured in complete growth medium consisting of high-glucose Dulbecco’s Modified Eagle Medium (DMEM) supplemented with 10% (v/v) heat-inactivated fetal bovine serum (FBS), and 1% (v/v) penicillin-streptomycin (100 U/mL penicillin and 100 μg/mL streptomycin). HepG2 cells were authenticated by ATCC using STR profiling and tested negative for mycoplasma contamination.

### Method details

#### Chemicals preparation

F-53B (CAS No. 73606-19-6, purity ≥98%) was obtained from Shanghai Maikun Chemical Co., Ltd. (Shanghai, China). A stock solution of F-53B (2 g/L) was prepared by dissolving the compound in dimethyl sulfoxide (DMSO, Sigma-Aldrich, St. Louis, USA) and subsequently diluting it with fish water (pH 7.5 ± 0.5, conductivity 500 ± 50 μS) or cell culture medium to achieve the desired working concentrations. To minimize the solvent effects, the final DMSO concentration in both the exposure and control groups was maintained below 0.5‰ (v/v). All other chemicals and solvents were of analytical or HPLC grade.

#### Zebrafish exposure

Three-month-old male wild-type adult zebrafish (AB strain; *n* = 320) were maintained in a zebrafish aquarium facility (Tecniplast Zebtec, Tecniplast, Buguggiate, Italy) at 27 ± 1 °C under a 14 h light/10 h dark photoperiod. After a 2-week acclimation period, the fish were assigned to two groups: ND_M and HFD_M. Fish in the ND_M group were fed freshly hatched artemia (60 mg/day/fish) twice daily for 8 weeks, whereas those in the HFD_M group were fed a high-fat diet consisting exclusively of egg yolk (100 mg/day/fish) and freshly hatched artemia (250 mg/day/fish), administered three times daily for 8 weeks to induce the MASLD model. After the 8-week modeling, fish in ND_M and HFD_M groups were not only further fed by ND and HFD, respectively, but also exposed to 0 (control), 0.25, 5, and 100 μg/L F-53B for 28 days in a semi-static system under the same rearing conditions, making a total of eight groups (ND_C, ND_0.25 μg/L, ND_5 μg/L, ND_100 μg/L, and HFD_C, HFD_0.25 μg/L, HFD_5 μg/L, HFD_100 μg/L). Notably, exposure concentrations were selected based on the previously reported concentrations of F-53B in contaminated water (ranging from 50.6 ng/L–112 μg/L).^4^^,^^49^ The 28-day exposure duration was chosen as it represents a well-established sub-chronic window in zebrafish toxicology and aligns with the experimental time frame used in comparable studies investigating the toxic effects by PFAS and their alternatives.^50^^,^^51^^,^^52^ For the exposure experiment, 28 fish from each group were equally distributed between two polypropylene tanks with 20 L of exposure solution. The exposure solutions were replaced daily. No dead fish or abnormal developmental phenotypes were observed at the end of the exposure period.

#### Zebrafish samples collection

At the end of the exposure period, the zebrafish were anesthetized using tricaine methanesulfonate (MS-222, Sigma-Aldrich, E10521) and dissected for tissue collection. To ensure representative sampling, fish were systematically collected in equal proportions from both holding tanks. For liver analysis, samples from each group were allocated as follows: three individual liver samples (*n* = 3, one fish per sample) were designated for F-53B quantification, six samples (*n* = 6, one fish per sample) for biochemical assays, six samples (*n* = 6, one fish per sample) for gene expression profiling, and five samples (*n* = 5, one fish per sample) for semi-quantitative PCR analysis. Additionally, eight liver samples (*n* = 8, one fish per sample) were reserved for histopathological evaluation, with four samples each processed for hematoxylin-eosin (HE) and Masson’s staining or Oil Red O (ORO) staining. To analyze serum parameters, including aspartate aminotransferase (AST) and alanine aminotransferase (ALT) activities, AST/ALT ratio, and albumin content, blood samples from four fish were pooled to constitute one biological replicate (*n* = 6 replicates per group).

#### MASLD zebrafish model verification

Three-month-old male wild-type zebrafish (AB strain; *n* = 160) were maintained in a Tecniplast Zebtec system under controlled conditions (27 ± 1 °C, 14:10 h light:dark cycle) for MASLD induction. Fish were fed with HFD consisting of 100 mg egg yolk and 250 mg freshly hatched artemia per fish daily, administered in three equal feedings for 8 weeks. Following dietary induction, 12 randomly selected fish were euthanized for validation. After taking photographs of the fish, their body length, body weight, and liver weight were recorded to calculate their body mass index (BMI) (*n* = 8) and relative liver weight (*n* = 4). BMI was calculated using the following formula: BMI = body weight/body length^2^ and relative liver weight = liver weight/body weight. Additionally, liver samples were processed for biochemical and histopathological analyses, including triglyceride (TG) and total cholesterol (T-CHO) quantification (*n* = 4), HE and Masson’s staining (*n* = 4), and ORO staining (*n* = 4), to confirm the successful establishment of MASLD.

#### L-FABP knockdown in HegG2 cells

L-FABP expression in HepG2 cells was knocked down using a commercial siRNA pool (L-FABP siRNA (h), Santa Cruz Biotechnology, sc-41243). Cells were seeded and transfected at 60–80% confluency using the recommended siRNA Transfection Reagent (Santa Cruz Biotechnology, sc-29528) and medium (Santa Cruz Biotechnology, sc-36868), with a non-targeting scrambled siRNA (Santa Cruz Biotechnology, sc-37007) used as a negative control. The siRNA-lipid complexes were incubated with the cells for 6–8 h before replacing the medium. Cells were harvested at 72 h post-transfection, and knockdown efficiency was confirmed by western blot analysis (*n* = 6, 3 biological replicates × 2 technical replicates) using an anti-L-FABP antibody (Santa Cruz Biotechnology, sc-271591).

#### Cell treatment

HepG2 cells were divided into four groups: (1) negative control siRNA (NC), (2) L-FABP knockdown (L-FABP KD), (3) NC with 5 mg/L F-53B (NC + F-53B), and (4) L-FABP KD with 5 mg/L F-53B (L-FABP KD + F-53B). Cells were transfected at 60–80% confluency using the specified siRNAs and transfection reagent. After the 6–8 h transfection period, the complexes were replaced with fresh complete growth medium. Cells were then allowed a 24-h recovery period (including the initial transfection time, totaling approximately 24 h post-seeding). Following this recovery, the medium was carefully replaced to initiate the 48-h F-53B exposure. Groups (3) and (4) received fresh medium containing 5 mg/L F-53B, while groups (1) and (2) received vehicle-control medium. All cells were harvested at the end of the 48-h exposure for determination of TG and the expression of interleukin-6 (IL-6) and transforming growth factor β1 (TGF-β1).

#### Quantification of F-53B in the exposure solution and zebrafish livers

F-53B extraction from the exposure solution and zebrafish liver was performed as previously described.^16^^,^^19^ F-53B was identified and quantified using an Agilent 6460 Triple Quadrupole LC-MS/MS System (Santa Clara, CA, USA) in the negative ESI mode with multiple reaction monitoring. Chromatographic separation was performed using an Eclipse Plus C18 column with mobile phases of 10 mM aqueous ammonium acetate and methanol. Data were quantified using an internal standard approach with ^13^C_4_-labeled L-PFOS (Wellington Laboratories, MPFOS). Notably, the measured concentrations of F-53B in the exposure solutions were comparable to the nominal concentrations (Table S3).

#### Histopathological examination

Liver samples (*n* = 8 per group, one sample per fish) were collected and fixed overnight in 4% paraformaldehyde for subsequent histopathological analysis. Among these, four liver samples were dehydrated, embedded in paraffin, and cut into 3–4 μm thick sections, which were subsequently subjected to HE staining for general histology assessment and Masson’s staining for hepatic fibrosis detection. The remaining four liver samples were cryo-embedded, and the frozen sections of 8–10 μm thickness were prepared for ORO staining to evaluate lipid accumulation. All the staining procedures were performed according to previously established protocols.^53^^,^^54^ Stained sections were examined under a light microscope (Olympus IX53). Hepatic vacuolation density, optical density in ORO staining, lymphocyte density, and percentage of fibrotic area were determined using the ImageJ 1.54 software (https://imagej.nih.gov/ij/).

#### Assessment of biochemical indicators

Liver samples were homogenized in 10 volumes of ice-cold phosphate buffer saline (PBS; 50 mM, pH 7.0) using an electric homogenizer. After centrifuging the homogenates at 3,000×*g* for 10 min at 4 °C, the supernatants were collected to determine the levels of TG, T-CHO, acetyl-CoA carboxylase (ACC), carnitine acyl-transferase-1 (CPT-1), free fatty acid (FFA), tumor necrosis factor-α (TNF-α), interleukin-1β (IL-1β), collagen I (ColIAI), hyaluronic acid (HA), and prothrombin time/factor II (PT/FII). Following the exposure period, HepG2 cells were collected, lysed, and the lysates were centrifuged to obtain supernatants for the determination of intracellular TG levels (*n* = 5). Additionally, serum samples were collected to determine AST and ALT activities, AST/ALT ratio, and albumin content. All biochemical indicators were determined using commercial reagent kits (Nanjing Jiancheng Bioengineering Institute, Nanjing, China), according to the manufacturer’s instructions.

#### Gene expression analysis

cDNA synthesis and quantitative real-time PCR (qRT-PCR) were performed as described previously.^16^ Specific primer sequences for target genes involved in fatty acid synthesis (*srebp1*, *srebp2*), fatty acid β-oxidation (*ppar-γ*), fatty acid esterification (*dgat2*), immune responses (*NF-κB*, *IκBα*), HA synthesis (*has2*, *has3*) and degradation (*hyal1*, *hyal2a*), and ColIAI degradation (*collagenase 3*) are listed in Table S4. Considering that *β*-actin was stable under our experimental conditions, it was chosen as the housekeeping gene for normalization. The relative expression levels of the target genes were analyzed using the 2^−ΔΔCt^ method.

#### Immunoblot

Total protein was extracted from harvested HepG2 cells using RIPA lysis buffer (Beyotime, P0013K), quantified by BCA assay (Beyotime, P0009), and denatured. Equal amounts of protein were separated by SDS-PAGE and transferred to PVDF membranes. After blocking, membranes were incubated overnight at 4 °C with the following primary antibodies: mouse anti-L-FABP (for knockdown validation, Santa Cruz Biotechnology, sc-271591), rabbit anti-IL-6 (Proteintech, 21865-1-AP), and rabbit anti-TGF-β1 (Proteintech, 81746-2-RR). GAPDH (Proteintech, 10494-1-AP) served as the loading control. Following incubation with appropriate HRP-conjugated secondary antibodies, protein bands were visualized using an ECL substrate and quantified by ImageJ 1.54. This protocol was used to confirm L-FABP knockdown efficiency and to assess the expression changes of IL-6 and TGF-β1 following F-53B exposure across all experimental groups (*n* = 6, 3 biological replicates × 2 technical replicates).

#### Semi-quantitative PCR

Due to the difficulty in obtaining consistent and quantifiable mRNA expression data for the regulatory gene *dbpb* via standard qRT-PCR, we employed semi-quantitative PCR as an alternative strategy. This method amplifies a specific promoter region of *dbpb* (from −2601 to −25 bp), where changes in amplification efficiency can serve as an indirect, comparative indicator of its potential transcriptional regulatory activity, which is relevant to albumin expression. Subsequently, semi-quantitative PCR was performed as follows: Total genomic DNA was extracted from the liver of fish in each group (*n* = 5, one fish per sample) using the DNAzol reagent (Thermo Fisher Scientific, 10503027), according to the manufacturer’s protocol. The concentration of the genomic DNA templates in each group was adjusted to ensure consistency with the intensity of the amplification product of *β*-actin to semi-quantitatively observe *dbpb* replication level. Specific primer sequences for *β*-actin and *dbpb* used for semi-quantitative PCR are listed in Table S5. Notably, the amplification amount on the promoter region located at −2601 bp to −25 bp in *dbpb* indicated the amplification efficiency of *dbpb*. PCR was performed with a 20 μL solution containing 250 nM of target gene specific primer, 0.2 mM dNTP, 0.5 U of TransTaq DNA polymerase (HiFi) (Transgen, AP131), and 100 ng genomic DNA templates. The PCR conditions were as follows: pre-denaturation at 95 °C for 5 min; 32 cycles of denaturation at 95 °C for 30 s, annealing at 60 °C for 30 s, and elongation at 72 °C for 90 s; elongation at 72 °C for 5 min.

### Quantification and statistical analysis

All data are expressed as mean ± standard error of the mean (SEM). Statistical analyses were performed using GraphPad Prism 9.0 (GraphPad Software, San Diego, CA, USA). Given the small sample sizes and potential deviations from normal distribution, non-parametric tests were employed for group comparisons. Comparisons involving multiple groups, such as effects of different F-53B concentrations within each diet group, were analyzed using the Kruskal-Wallis test. If a significant overall difference was detected, post-hoc pairwise comparisons were performed using Dunn’s test with Bonferroni correction. For comparisons between two specific groups, including the initial model validation (ND_M vs. HFD_M), the evaluation of ND and HFD groups under the same F-53B concentration, the assessment of L-FABP knockdown efficiency in HepG2 cells, and the analyses of TG, IL-6, and TGF-β1 expression between the following paired groups of NC + F-53B vs. NC, L-FABP KD + F-53B vs. L-FABP KD, and (NC + F-53B/NC) vs. (L-FABP KD + F-53B/L-FABP KD), the Mann-Whitney U test was performed. Statistical significance was defined as *p* < 0.05.
