# Supplementary material for: F-53B exposure accelerates progression from preexisting fatty liver to non-alcoholic steatohepatitis and hepatic fibrosis
Source: iScience. 2026 Apr 10;29(5):115675. doi: 10.1016/j.isci.2026.115675 (PMC13127485; doi:10.1016/j.isci.2026.115675)
Supplement: Document S1. Figures S1–S3 and Tables S1–S5 [file mmc1.pdf]

iScience, Volume *29*

Supplemental information

F-53B exposure accelerates progression from preexisting fatty liver to non-alcoholic steatohepatitis and hepatic fibrosis

Chunhua Hu, Liu Wu, Zehui Zhang, Yu Liu, Huihui Yang, Jian Zhou, Yongming Wu, and Qiyu Wang

Figure S1. L-FABP knock down efficiency assessment.

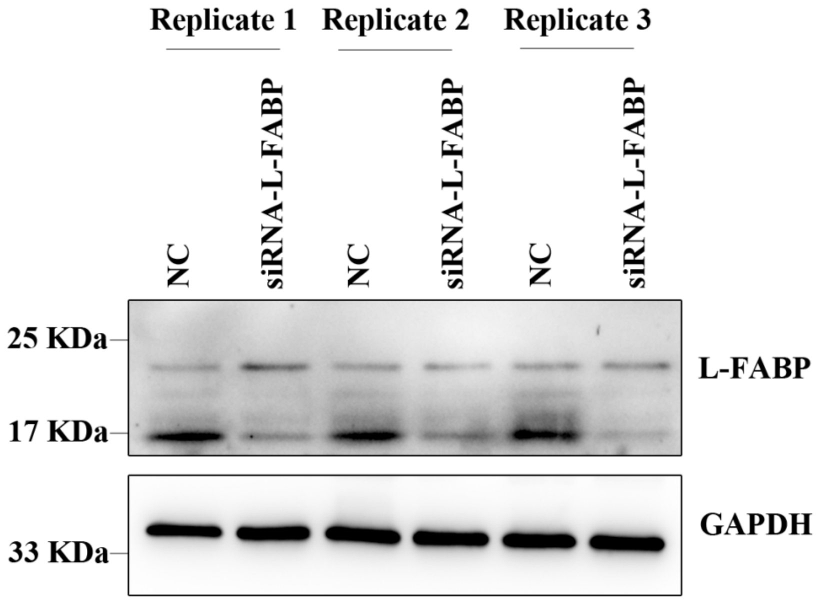

Figure S2. Expression level of IL-6 in HepG2 cells following different treatments.

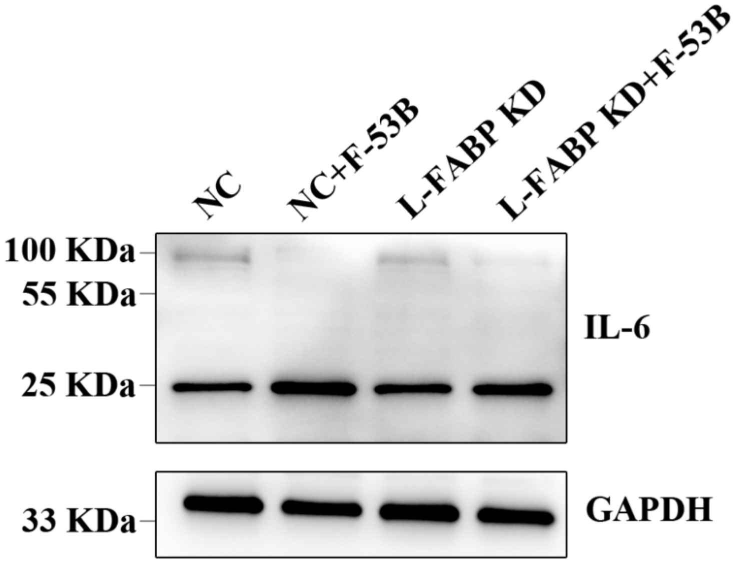

Figure S3. Expression level of TGF-β1 in HepG2 cells following different treatments.

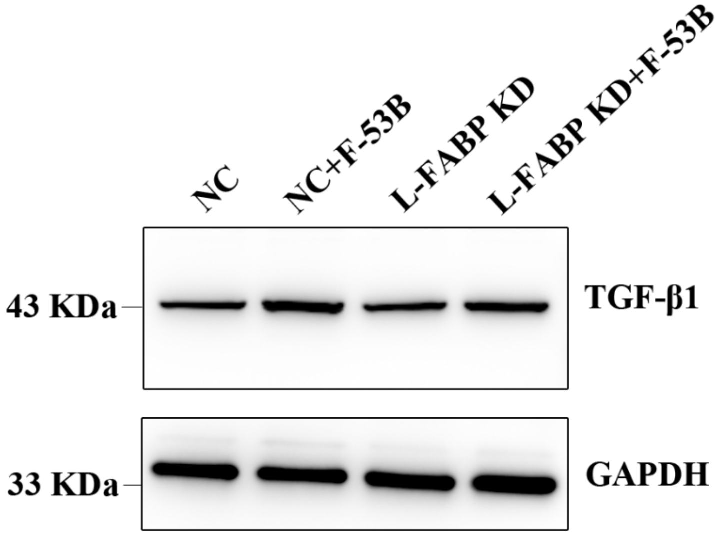

Table S1. Summary of key biomarker changes related to MASH following F-53B exposure.

Biomarker	Comparison Group	Mean ± SD (Control)	Mean ± SD (Treated)	Fold Change vs. Control	*p*-value
TNF-α	ND_C vs. ND_100 μg/L	0.88 ± 0.11	1.01 ± 0.07	1.15	< 0.05
TNF-α	HFD_C vs. HFD_0.25 μg/L	0.94 ± 0.13	1.18 ± 0.13	1.22	< 0.05
TNF-α	HFD_C vs. HFD_5 μg/L	0.94 ± 0.13	1.13 ± 0.09	1.17	< 0.05
TNF-α	HFD_C vs. HFD_100 μg/L	0.94 ± 0.13	1.25 ± 0.20	1.32	< 0.05
TNF-α	ND_0.25 μg/L vs. HFD_0.25 μg/L	0.96 ± 0.10	1.18 ± 0.13	1.19	< 0.05
TNF-α	ND_5 μg/L vs. HFD_5 μg/L	0.96 ± 0.09	1.13 ± 0.09	1.15	< 0.05
TNF-α	ND_100 μg/L vs. HFD_100 μg/L	1.01 ± 0.07	1.25 ± 0.20	1.23	< 0.05
IL-1β	ND_C vs. ND_100 μg/L	0.37 ± 0.52	1.7 ± 0.73	4.65	< 0.05
IL-1β	HFD_C vs. HFD_0.25 μg/L	0.27 ± 0.25	1.42 ± 0.71	5.37	< 0.05
IL-1β	HFD_C vs. HFD_100 μg/L	0.27 ± 0.25	1.39 ± 0.81	5.23	< 0.05
Albumin	HFD_C vs. HFD_0.25 μg/L	16.43 ± 2.36	22.10 ± 0.95	1.34	< 0.01
Albumin	HFD_C vs. HFD_5 μg/L	16.43 ± 2.36	22.97 ± 2.83	1.40	< 0.01
Albumin	HFD_C vs. HFD_100 μg/L	16.43 ± 2.36	20.97 ± 1.32	1.28	< 0.01
Albumin	ND_0.25 μg/L vs. HFD_0.25 μg/L	12.32 ± 1.25	22.10 ± 0.95	1.79	< 0.001
Albumin	ND_5 μg/L vs. HFD_5 μg/L	12.12 ± 0.95	22.97 ± 2.83	1.89	< 0.01
Albumin	ND_100 μg/L vs. HFD_100 μg/L	12.25 ± 1.36	20.97 ± 1.32	1.71	< 0.001

[1] Control and Treated refer to the specific group comparisons shown.

[2] Fold Change was calculated as (Mean_Treated / Mean_Control).

Table S2. Summary of key biomarker changes related to fibrosis following F-53B exposure.

Biomarker	Comparison Group	Mean ± SD (Control)	Mean ± SD (Treated)	Fold Change vs. Control	*p*-value
ColIAI	ND_C vs. ND_100 μg/L	0.14 ± 0.02	0.08 ± 0.01	0.57	< 0.001
ColIAI	HFD_C vs. HFD_0.25 μg/L	0.12 ± 0.03	0.08 ± 0.01	0.70	< 0.05
ColIAI	HFD_C vs. HFD_5 μg/L	0.12 ± 0.03	0.09 ± 0.01	0.75	< 0.05
ColIAI	HFD_C vs. HFD_100 μg/L	0.12 ± 0.03	0.08 ± 0.02	0.67	< 0.05
ColIAI	ND_0.25 μg/L vs. HFD_0.25 μg/L	0.16 ± 0.02	0.08 ± 0.01	0.52	< 0.001
ColIAI	ND_5 μg/L vs. HFD_5 μg/L	0.14 ± 0.02	0.09 ± 0.01	0.61	< 0.01
HA	ND_C vs. ND_100 μg/L	0.11 ± 0.01	0.07 ± 0.01	0.67	< 0.01
HA	HFD_C vs. HFD_0.25 μg/L	0.10 ± 0.02	0.06 ± 0.01	0.65	< 0.01
HA	HFD_C vs. HFD_5 μg/L	0.10 ± 0.02	0.07 ± 0.01	0.75	< 0.05
HA	HFD_C vs. HFD_100 μg/L	0.10 ± 0.02	0.07 ± 0.02	0.68	< 0.05
HA	ND_0.25 μg/L vs. HFD_0.25 μg/L	0.12 ± 0.01	0.06 ± 0.01	0.55	< 0.001
HA	ND_5 μg/L vs. HFD_5 μg/L	0.10 ± 0.01	0.07 ± 0.01	0.71	< 0.05
PT/FII	ND_C vs. ND_100 μg/L	0.57 ± 0.08	0.36 ± 0.05	0.63	< 0.01
PT/FII	HFD_C vs. HFD_0.25 μg/L	0.53 ± 0.10	0.35 ± 0.04	0.65	< 0.01
PT/FII	HFD_C vs. HFD_5 μg/L	0.53 ± 0.10	0.42 ± 0.03	0.79	< 0.05
PT/FII	HFD_C vs. HFD_100 μg/L	0.53 ± 0.10	0.39 ± 0.09	0.74	< 0.05
PT/FII	ND_0.25 μg/L vs. HFD_0.25 μg/L	0.63 ± 0.08	0.35 ± 0.04	0.55	< 0.001

[1] Control and Treated refer to the specific group comparisons shown.

[2] Fold Change was calculated as (Mean_Treated / Mean_Control).

Table S3. Quantification of F-53B in the exposure solutions.

	Nominal concentration (μg/L)	Mean measured concentration (μg/L) ± SEM	
		T_0	T_{24}
ND_C	0.000	non-detected	non-detected
ND_0.25 μg/L	0.250	0.211±0.006	0.193±0.007
ND_5 μg/L	5.000	4.021±0.102	4.102±0.089
ND_100 μg/L	100.000	79.256±2.428	76.586±2.043
HFD_ C	0.000	non-detected	non-detected
HFD_0.25 μg/L	0.250	0.198±0.003	0.186±0.005
HFD_5 μg/L	5.000	4.212±0.143	3.998±0.108
HFD_100 μg/L	100.000	80.234±3.024	77.433±2.763

Table S4. The primer sequences for qRT-PCR.

Gene name	Accession NO.	Forward Primer	Reversed Primer
β-actin	NM_131031.2	CGAGCAGGAGATGGGAACC	CAACGGAAACGCTCATTGC
srebp1	NM_001105129.1	CTAACCGACAGCCAAGTGAA	AGACGACAACAACAACAACAAC
srebp2	NM_001089466.1	AGGAGGAGTGGTGAAGGA	GTTGATGGAGGAGCGGTAG
ppar-γ	NM_131467.1	GGAACTGGAGGAGCTGGAGGAC	CGATGCCTGATATGCTGCTGTAGTC
dgat2	NM_001030196.1	ACGCATAACCTGCTTCCC	TCCTGTGGCTTCTGTCCC
nfkb2	NM_001001840.3	CATATGTCCCACACAATCAAGAC	AGCCACCATAATGATCTGGAA
IkBα	NM_00112 3265.1	CAAACTGGTGGTTCAAGCCG	CACTCACTGGACTGCGAACT
has2	AF190742.1	CCGCCTTCAAAGCCCTGGGA	TCCGCTGATACATTGAACGCACCC
has3	XM_068214710.1	GGGGTGGCAAGAGGGAAGTGATGTA	CCCGCTCCACATTGAAGGCCAT
hyal1	XM_068221787.1	CGGTGTCGTGCTGTGGGGAGAT	CGGCCAAGGGTGACGTCCAGATA
hyal2a	XM_009302730.4	GCCGCCCTACGTATGCCAATGAG	GCCCAGTGGACCGCGCAAGTA
collagenase 3	NM_001290479.1	GCGCCGCTTGCTGGAGGAGA	CGGGCTCTGTGCAGGCTGTTTC

Table S5. The primer sequences used for semi-quantitative PCR analysis.

Gene name	Accession NO.	Forward Primer	Reversed Primer
β-actin	NM_131031.2	CGAGCAGGAGATGGGAACC	CAACGGAAACGCTCATTGC
dbpb	AL928695.23	TGGAGTTCTACAGCTCTGGGAAAAATAAC	GATGCTTGCGTGGTTCGATACTATTGT
